# RNF20 dual regulation of MDA5 signaling to maintain immune homeostasis in chickens

**DOI:** 10.1128/jvi.02008-24

**Published:** 2025-02-25

**Authors:** Jie Wang, Qiuju Liu, Kehui Zhang, Shurui Zhao, Qi Shao, Feiyu Fu, Jingjiao Ma, Zhaofei Wang, Ya-xian Yan, Hengan Wang, Jianhe Sun, Yuqiang Cheng

**Affiliations:** 1 Shanghai Key Laboratory of Veterinary Biotechnology, School of Agriculture and Biology, Shanghai Jiao Tong University 596493 , Shanghai, China; University Medical Center Freiburg, Freiburg, Germany

**Keywords:** chicken, RNF20, MDA5, type I interferon, innate immunity, RNA virus

## Abstract

RIG-I and MDA5, known as the RIG-I-like receptors (RLRs), play a pivotal role in inducing antiviral responses to RNA viral infections. While chickens lack RIG-I, they possess a functionally enhanced MDA5 that recognizes pathogens and regulates immunity, underscoring the critical role of MDA5 in maintaining immune homeostasis in chickens. However, the precise mechanisms governing the expression and optimal activation of MDA5 remain unclear. Here, we reveal that the chicken E3 ubiquitin ligase RNF20 is essential for modulating MDA5-mediated innate immune homeostasis. Transcriptome sequencing analysis revealed that RNA viral infection of DF-1 cells significantly upregulated the expression of chicken RNF20. Overexpression of RNF20 markedly suppresses the expression of chicken innate immunity-related genes, while RNF20 knockout leads to immune deficiency both *in vivo* and *in vitro*. Mechanistically, RNF20 is located in the nucleus, where it maintains the basic expression and regulates the inducible expression of MDA5 to establish immune defense during the early infection phase. In the late phase, RNF20 translocates to the cytoplasm, where it facilitates the K27- and K48-linked polyubiquitination and subsequent degradation of MDA5, thereby preventing immune overstimulation. Overall, this study establishes RNF20 as an important E3 ubiquitin ligase that maintains chicken innate immune homeostasis.

**IMPORTANCE:**

Chicken MDA5 is an important RNA viral sensor for initiating the antiviral innate immune response. The protein level of MDA5 must be tightly regulated to maintain antiviral innate immune homeostasis. In this study, we demonstrate that the E3 ubiquitin ligase RNF20 precisely regulates MDA5 protein stabilization through nucleoplasmic translocation. Specifically, in uninfected and during early infection, RNF20 regulates MDA5 transcription in the nucleus. While in the late stages of infection, RNF20 translocates out of the nucleus and catalyzes the ubiquitinated degradation of MDA5. Thus, RNF20 is important in regulating chicken antiviral innate immune homeostasis.

## INTRODUCTION

The innate immune system serves as the initial defense against invading pathogens, providing immediate protection against a broad spectrum of pathogens, including bacteria, viruses, fungi, and parasites (1). These pathogens possess conserved pathogen-associated molecular patterns (PAMPs) that are rapidly detected and recognized by various pattern-recognition receptors (PRRs) (2). PRRs subsequently initiate a cascade of reactions, releasing cytokines, such as type I interferons (IFNs) and pro-inflammatory cytokines, to control pathogenic microbial infections (3).

The host’s PRRs primarily include Toll-like receptors (TLRs), retinoic acid-inducible gene I (RIG-I)-like receptors (RLRs), and nucleotide-binding oligomerization domain (NOD)-like receptors (NLRs) (4). Among them, RLRs, including RIG-I and melanoma differentiation-associated gene 5 (MDA5), are key PRRs involved in RNA virus recognition (5). Notably, chickens lack a gene homologous to mammalian RIG-I; instead, chicken MDA5 (chMDA5), structurally similar to RIG-I, functionally compensates for this absence (6). Therefore, the expression and activation of chMDA5 are crucial for initiating the innate immune response and maintaining immune homeostasis. Although numerous molecules that regulate MDA5 activation have been identified in mammals, significant differences exist between the innate immune systems of chickens and mammals (7, 8). The regulatory mechanisms governing chMDA5 activation remain unclear.

Protein post-translational modifications (PTMs) are essential for regulating immunity by influencing the activation, survival, proliferation, differentiation, and migration of immune cells (9). Among these PTMs, ubiquitination regulates the activation of PRRs and downstream signaling molecules, thereby influencing PRR-dependent innate immune responses (10, 11). Typically, K63-linked polyubiquitin chains activate protein effector functions, while K27 and K48-linked polyubiquitin chains typically mark target proteins for proteasomal degradation (12, 13). The E3 ubiquitin ligases determine substrate specificity for ubiquitination process (14). For example, TRIM25-catalyzed K63-linked polyubiquitination of RIG-I is essential for RIG-I activation and IFN production (15). Conversely, TRIM40 catalyzes the K27 and K48-linked ubiquitination and degradation of MDA5 and RIG-I, effectively attenuating the innate immune response to prevent overstimulation (16). This suggests that E3 ubiquitin ligase plays a significant role in regulating innate immune homeostasis.

E3 ubiquitin ligase Ring Finger Protein 20 (RNF20), a member of the RING protein family and homolog to yeast BRE1, contains a really interesting new gene (RING) domain and multiple coiled-coil domains. The number of coiled-coil domains increases from yeast to higher animals, leading to more complex functions (17). Research shows that RNF20 is primarily localized in the nucleus, where it regulates DNA damage repair, meiosis, cell proliferation, and T-cell differentiation (18–20). Furthermore, RNF20 also functions as a transcriptional co-activator, regulating the expression of genes involved in lipid synthesis and immune responses (21–23). Moreover, transcriptome sequencing revealed that RNF20 expression is upregulated in chickens following infection with highly pathogenic avian influenza. Notably, RNF20 expression is also upregulated during eggshell formation in chickens (24, 25). However, the role of RNF20 in regulating innate immune responses in chickens remains largely unclear.

In this study, we found that RNF20 dually regulates the chicken MDA5-mediated innate immune response to maintain immune homeostasis. Transcriptome sequencing and library screening revealed that RNF20 regulates the chicken innate immune response. Overexpression and knockdown experiments showed that RNF20 negatively regulates virus-induced innate immune responses in chicken. However, RNF20 knockout results in immunodeficiency both *in vitro* and *in vivo*. Immunofluorescence staining revealed that RNF20 was localized in the nucleus. Upon viral infection, RNF20 translocates from the nucleus to the cytoplasm, where it differentially regulates MDA5. Specifically, RNF20 regulates MDA5 expression in the nucleus to maintain immune defense, while in the cytoplasm, it catalyzes K27 and K48-linked polyubiquitination and degradation of MDA5. This process attenuates the MDA5-induced immune response and prevents excessive immune activation. In conclusion, this study identifies RNF20 as an important E3 ubiquitin ligase involved in regulating chicken innate immune homeostasis.

## RESULTS

### RNF20 is involved in the regulation of chicken innate immunity response

**Fig 1:**
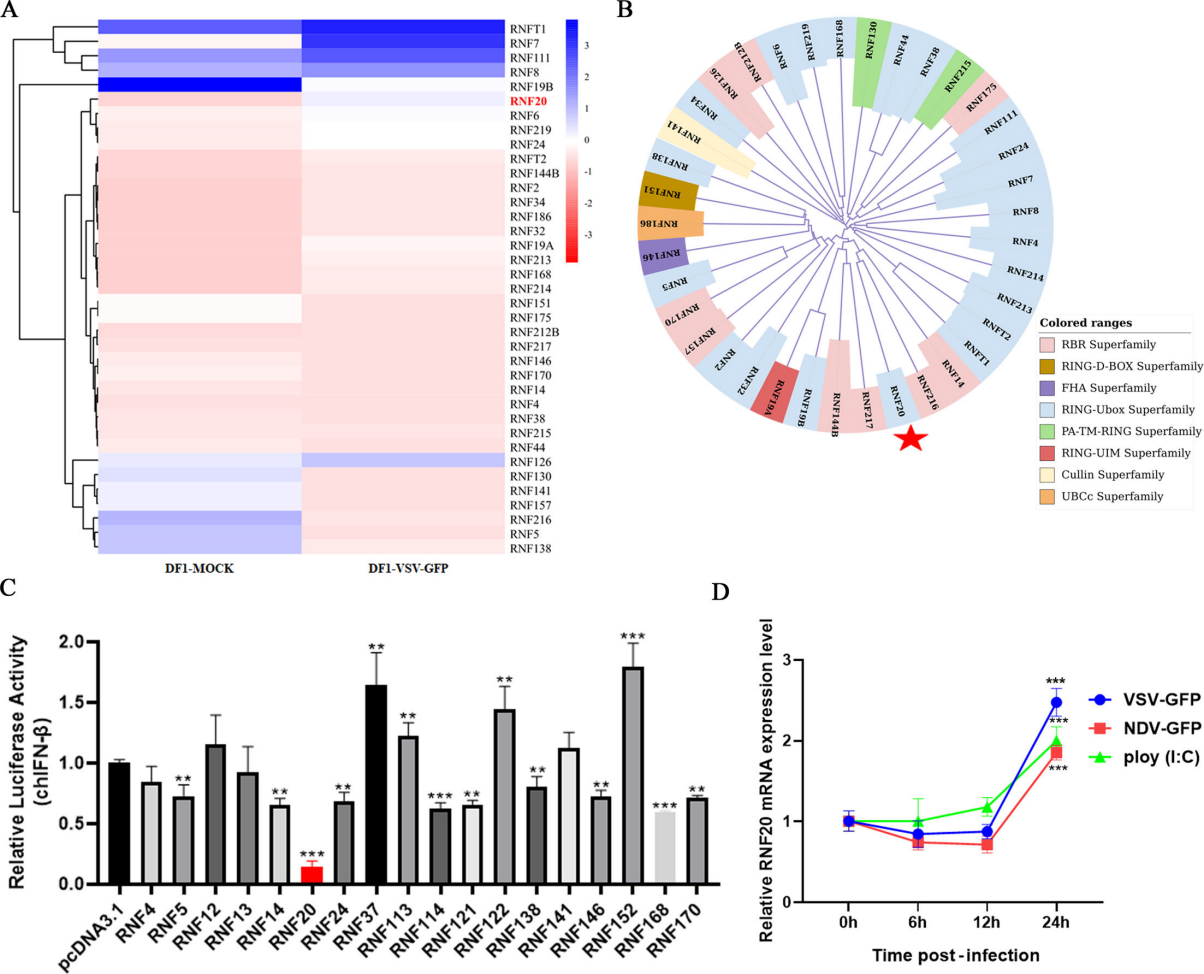
Chicken RNF20 is involved in the regulation of innate immunity. (A) Heat map analysis of differentially expressed RNF family genes by transcriptome sequencing of DF-1 cells infected with VSV-GFP (multiplicity of infection [MOI] =1) for 24 h. (**B**) Phylogenetic tree classifying differentially expressed RNF family members. (**C)** DF-1 cells were transiently transfected with expression plasmids for RNF family proteins or control plasmids (250 ng/well), pRL-TK (60 ng/well), and IFN-β Luc (120 ng/well). After 24 h, the cells were infected with VSV-GFP (MOI = 1) for 12 h, followed by analysis using a dual luciferase reporter assay. (**D)** DF-1 cells were transfected with poly (I: C) (0.5 mg/mL) or infected with NDV-GFP or VSV-GFP (multiplicity of infection [MOI] =1), and the cells were harvested at the indicated time points for detecting RNF20 mRNA expression. Data of three independent experiments are shown as mean ± SD. *P < 0.05, **P < 0.01, and ***P < 0.001 are considered statistically significant.

To investigate the E3 ubiquitin ligases involved in regulating the innate immune response in chickens, we conducted transcriptome sequencing to analyze the differential expression of RNF family members following vesicular stomatitis virus (VSV-GFP) infection in DF-1 chicken cells. It was found that VSV-GFP infection significantly alters the expression of RNF family members (Fig. 1A). Phylogenetic tree analysis and functional domain classification of these differentially expressed RNF family members revealed they can be divided into three branches, primarily belonging to the RING-Ubox and RBR superfamilies (Fig. 1B). This suggests that these two superfamilies of RNF proteins play an important role in chicken innate immunity.

To further identify RNF family members involved in regulating the innate immune response, we screened several RNF family proteins using a transcriptome library. We found that RNF20 exhibited the most significant inhibitory effect on the IFN-β promoter compared with other members (Fig. 1C). Additionally, infections with VSV-GFP, Newcastle disease virus (NDV-GFP), and the RNA virus nucleic acid mimic poly (I: C) result in a significant upregulation of RNF20 expression in DF-1 cells (Fig. 1D). These results suggest that RNF20 may play a critical role in the innate immune response of chickens.

### Chicken RNF20 negatively regulates innate immune response

**Fig 2:**
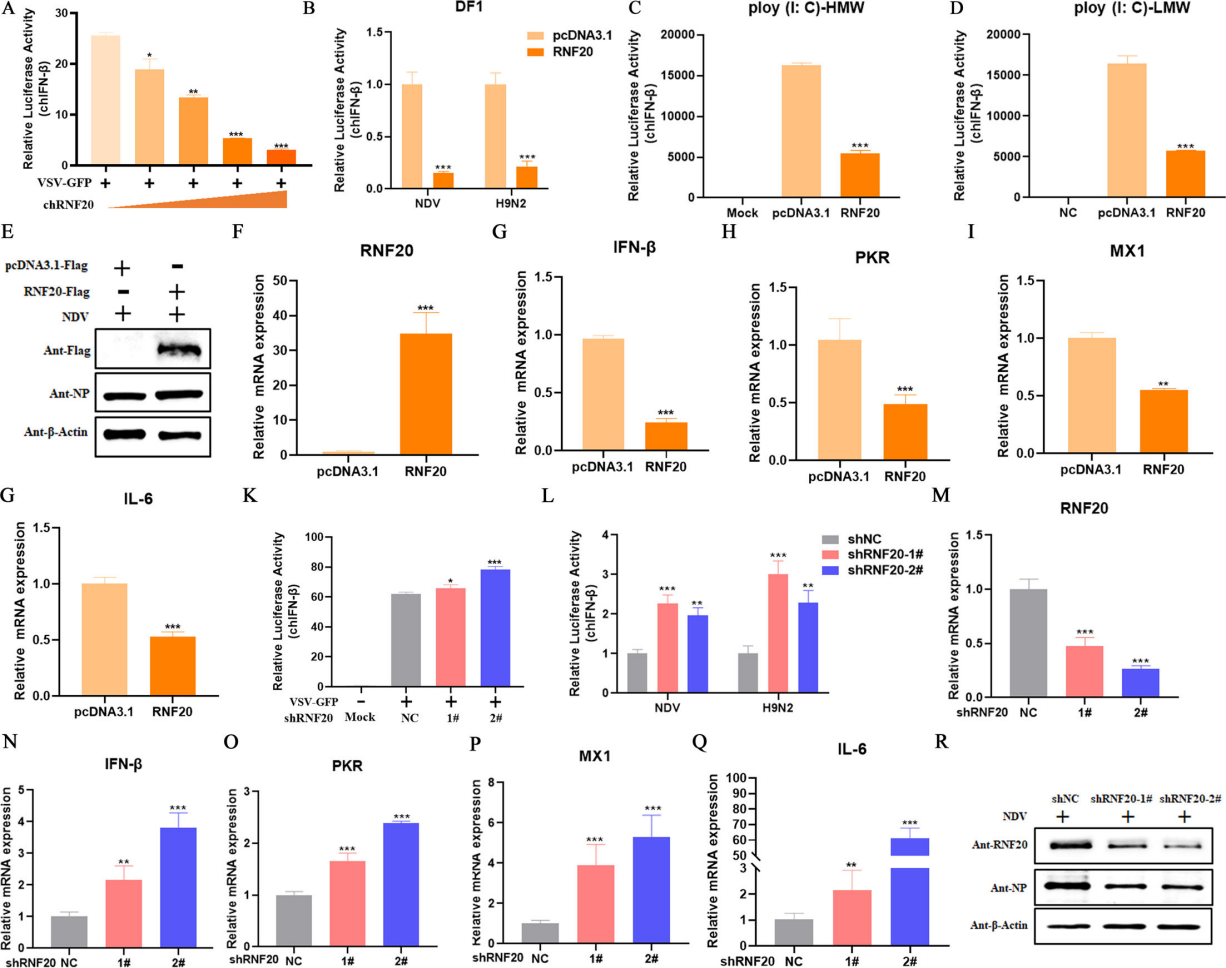
Chicken RNF20 negatively regulates innate immune response. (A and B) DF-1 cells were transiently transfected with RNF20 expression plasmids (0, 50, 100, 200, 300 ng/well), pRL-TK (60 ng/well), and IFN-β Luc (120 ng/well). After 24 h, the cells were infected with VSV-GFP, NDV, or H9N2 (multiplicity of infection [MOI] =1) for 12 h and analyzed by dual luciferase reporter. (C and D) DF-1 cells were transiently transfected with RNF20 expression plasmids (250 ng/well) or control plasmid (250 ng/well), poly (I: C)-HWM or poly (I: C)-LWM, pRL-TK (60 ng/well), and IFN-β Luc (120 ng/well). After 24 h, cells were analyzed by dual luciferase reporter assay. (**E)** DF-1 cells were transiently transfected with RNF20 expression plasmids (500 ng/well) or control plasmids (500 ng/well). After infection with NDV (multiplicity of infection [MOI] =1) for 24 h, the cells were harvested for detecting RNF20, NDV-NP, and β-actin protein expression. (**F-G)** DF-1 cells were transiently transfected with RNF20 expression plasmids (500 ng/well) or control plasmids (500 ng/well). After infection with NDV (multiplicity of infection [MOI] =1) for 12 h, the cells were harvested for detecting mRNA expression of RNF20, IFN-β, PKR, MX1, and IL-6. (K and L) DF-1 cells were transiently transfected with RNF20 interfering plasmids (250 ng/well) or control plasmid (250 ng/well), pRL-TK (60 ng/well), and IFN-β Luc (120 ng/well). After 24 h, the cells were infected with VSV-GFP, NDV, or H9N2 (multiplicity of infection [MOI] =1) for 12 h and analyzed by dual luciferase reporter. (**M-Q)** DF-1 cells were transiently transfected with RNF20 interfering plasmids (1,000 ng/well) or control plasmids (1,000 ng/well). After infection with VSV-GFP (MOI = 1) for 12 h, the cells were harvested for detecting mRNA expression of RNF20, IFN-β, PKR, MX1, and IL-6.** (R)** DF-1 cells were transiently transfected with RNF20 interfering plasmids (1,000 ng/well) or control plasmids (1,000 ng/well). After infection with NDV (MOI = 1) for 12 h, the cells were harvested to detect RNF20, NDV-NP, and β-actin protein expression. Data from three independent experiments are shown as mean ± SD. *P < 0.05, **P < 0.01, and ***P < 0.001 are considered statistically significant.

To elucidate the role of RNF20 in innate immune responses, we overexpressed RNF20 in DF-1 cells and subsequently stimulated them with RNA viruses, such as VSV-GFP, NDV, or H9N2, as well as RNA virus mimics poly (I: C). The results demonstrate that RNF20 significantly inhibits the activation of the IFN-β promoter induced by the virus, high-molecular-weight (HMW) poly (I: C), and low molecular weight (LMW) (Fig. 2A through D). Western blot analysis of the NDV virus replication revealed that overexpression of RNF20 significantly increased NDV NP protein expression (Fig. 2E). qPCR analysis also revealed that RNF20 markedly reduces the expression of innate immunity-related genes, including IFN-β, PKR, and MX1, as well as the inflammation-related gene IL-6 (Fig. 2F and G). In contrast, RNF20 knockdown resulted in a significant enhancement of IFN-β promoter activation induced by VSV-GFP, NDV, and H9N2 (Fig. 2K and L) and significantly promoted the expression of innate immunity and inflammation-related genes, as well as significantly inhibiting viral replication (Fig. 2M through R). These results indicate that chicken RNF20 negatively regulates innate immune responses.

### RNF20 negative regulation of innate immune response is conserved among species

**Fig 3:**
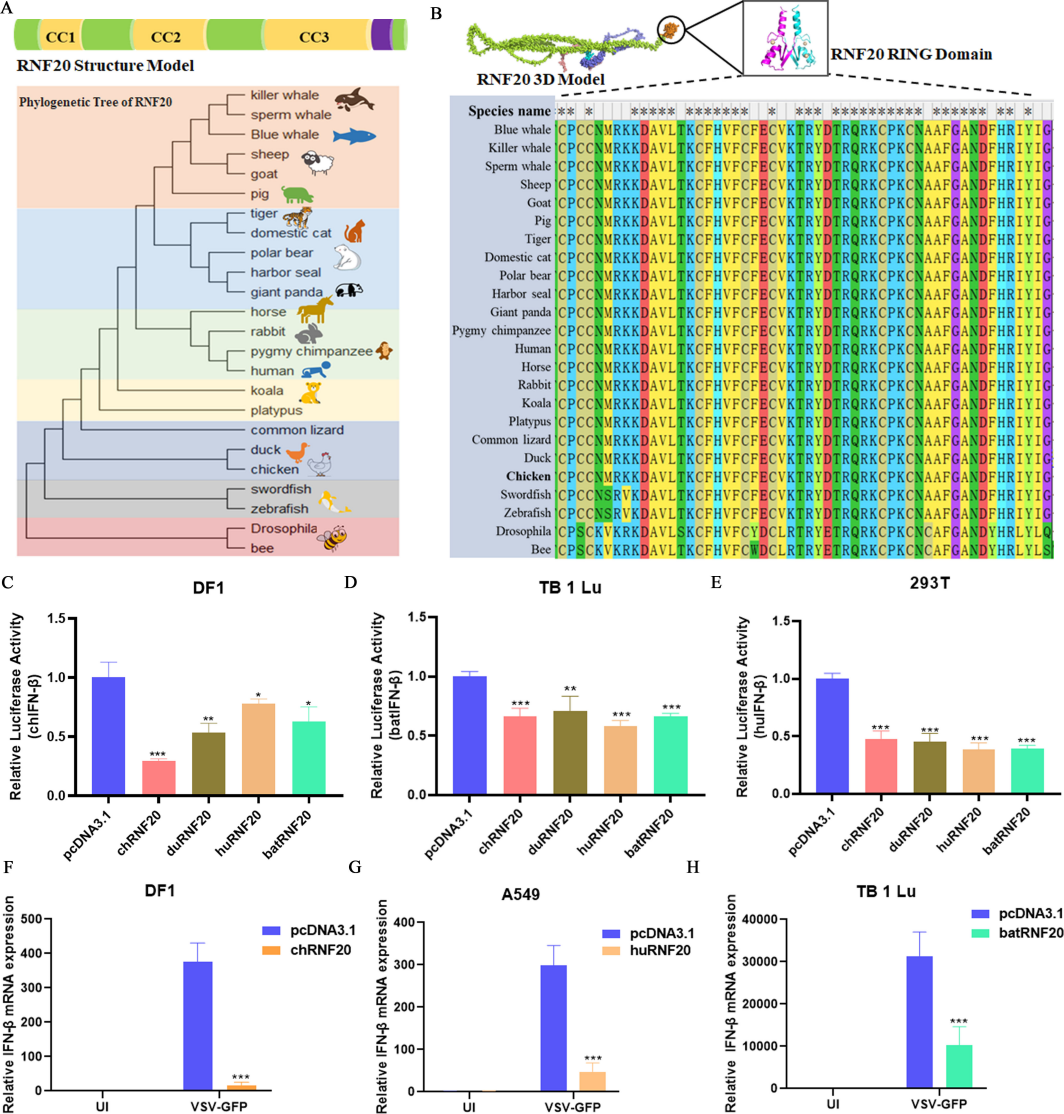
RNF20 negative regulation of innate immune response is conserved among species. (A). Phylogenetic tree analyses of RNF20 amino acid sequence. (**B)** Alignment of the amino acid sequence of the RNF20 RING domain across species using the Mega 11. (**C-E)** DF-1, TB 1 Lu, or 293T cells were transiently transfected with RNF20 expression plasmids from chicken, duck, human, or bat, or control plasmid (250 ng/well), pRL-TK (60 ng/well), and IFN-β Luc (120 ng/well). After 24 h, the cells were infected with NDV (multiplicity of infection [MOI] =1) for 12 h and analyzed by dual luciferase reporter. (**F-H)** DF-1, A549, and TB 1 Lu cells transiently transfected with RNF20 expression plasmid or control plasmid (500 ng/well). After unaffecting (UI) or infecting with VSV-GFP for 24 h, the cells were harvested for detecting IFN-β mRNA expression. Data from three independent experiments are shown as mean ± SD. *P < 0.05, **P < 0.01, and ***P < 0.001 are considered statistically significant.

To further investigate the function of RNF20, we conducted a phylogenetic tree analysis of its amino acid sequence. This analysis revealed that the RNF20 amino acid sequence is species-specific, with distinct branches formed by insects, fish, birds, and mammals (Fig. 3A). However, RNF20 from different species consists of three coiled-coil domains and one RING domain. Notably, the N terminus of RNF20 from various species contains a conserved RING domain (Fig. 3B), suggesting that the function of RNF20 may be conserved across species.

To explore the conservation of RNF20 function across species, we cloned RNF20 from chicken, duck, human, and bat and overexpressed them in chicken DF-1 cells, human 293T cells, and bat TB 1 Lu cells, respectively. We found that RNF20 from different species significantly inhibited virus-induced IFN-β promoter activity (Fig. 3C through E). Additionally, the overexpression of RNF20 from chicken, humans, and bats notably suppressed VSV-GFP-induced IFN-β mRNA expression in cells derived from chicken, humans, and bats (Fig. 3F through H). These findings suggest that the function of RNF20 is highly conserved across species.

### RNF20 negatively regulates innate immune responses by targeting MDA5

**Fig 4:**
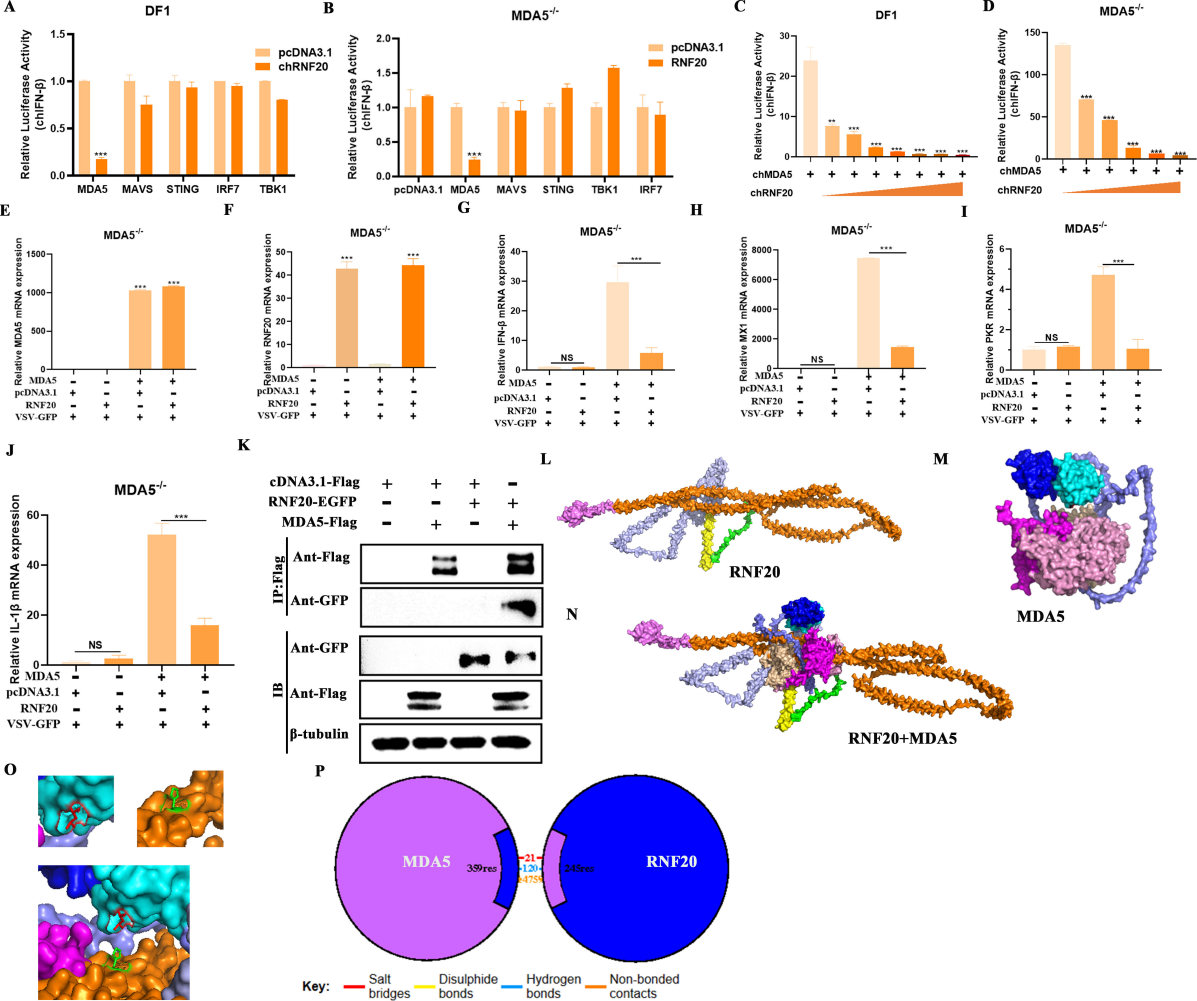
RNF20 negatively regulates innate immune responses by targeting MDA5. (A and B) DF-1 wild type or MDA5^−/−^ cells were transiently transfected with the indicated RLR adaptor plasmids (100 ng/well) along with an RNF20-expressing plasmid or control plasmid (150 ng/well), pRL-TK (60 ng/well), and IFN-β Luc (120 ng/well). After 24 h, the cells were infected with NDV (multiplicity of infection [MOI] =1) for 12 h and analyzed by dual luciferase reporter. (C and D) DF-1 wild cells or DF-1 MDA5^−/−^ cells were transiently transfected with MDA5 expressing plasmid (100 ng/well), along with increasing amounts (wedge) of an RNF20-expressing plasmid (0, 50, 100, 150, 200, 250, 300, and 400 ng/well), pRL-TK (60 ng/well), and IFN-β Luc (120 ng/well). After 24 h, the cells were infected with NDV (multiplicity of infection [MOI] =1) for 12 h and analyzed for luciferase activity using a dual luciferase reporter. (**E-J)** DF-1 MDA5^−/−^ cells were transiently transfected with RNF20 or MDA5 expression plasmid (500 ng/well). After infection with NDV for 24 h, the cells were harvested to detect MDA5, RNF20, IFN-β, MX1, PKR, and IL-1β mRNA expression. (**K)** HEK293T cells were transfected with RNF20-EGFP and MDA5-Flag expression plasmid (1000 ng/well) in six-well plates. Co-immunoprecipitation (Co-IP) with cell lysates was performed after 12 h using Flag antibodies, followed by immunoblotting (IB). (L and M) Alphafold2 were used to predict the three-dimensional structure of chicken RNF20 or chicken MDA5. (**N)** PyMOL was used to perform molecular docking on the three-dimensional structures of RNF20 and MDA5. (**O)** Display of interaction sites between RNF20 and MDA5. (**P)** PBDsum online analysis of interaction types between RNF20 and MDA5 (https://www.ebi.ac.uk/thornton-srv/databases/pdbsum/). Data from three independent experiments are shown as mean ± SD. *P < 0.05, **P < 0.01, and ***P < 0.001 are considered statistically significant.

The RLR signaling pathway plays a crucial role in RNA virus infection. To investigate the mechanism by which RNF20 negatively regulates the innate immune response, we co-transduced RNF20 with key molecules of the chicken RLR signaling pathway. We found that RNF20 significantly inhibited MDA5-induced IFN-β promoter activation, no inhibition was observed with MAVS, STING, TBK1, or IRF7 (Fig. 4A). This suggests that RNF20 may specifically target chicken MDA5. To further demonstrate this, we co-transfected RNF20 and key proteins of the RLR signaling pathway into DF-1 MDA5^−/−^ cells. RNF20 lost its inhibitory effect in MDA5^−/−^ cells but regained it upon reintroduction of MDA5. The introduction of other molecules had no effect, indicating that RNF20 specifically inhibits MDA5-induced IFN-β promoter activation (Fig. 4B).

Additionally, we co-transfected MDA5 and RNF20 into DF-1 wild-type and MDA5^−/−^ cells, demonstrating that RNF20 inhibited MDA5-mediated IFN-β promoter activation in a concentration-dependent manner (Fig. 4C and D). qPCR analysis showed that RNF20 lost its effect on genes related to innate immunity, including IFN-β, PKR, MX1, and the inflammatory factor gene IL-1β in MDA5^−/−^ cells. However, after reintroducing MDA5, RNF20 restored its inhibitory effect on these genes (Fig. 4E through J). These results suggest that RNF20 inhibits the MDA5-mediated innate immune response. Co-immunoprecipitation confirmed the physical interaction between RNF20 and MDA5 (Fig. 4K). Next, we predicted the protein structures of RNF20 and MDA5 by using AlphaFold2 and performed protein–protein docking analysis with PyMOL. The results indicate that the third coiled-coil domain of RNF20 interacts with the CARD domain of MDA5 (Fig. 4L through O), with PBDsum analysis revealing hydrogen bonds and salt bridges between MDA5 and RNF20 (Fig. 4P). These findings indicate that RNF20 negatively regulates the innate immune response through interaction with MDA5.

### RNF20 degrades MDA5 via the ubiquitin–proteasome pathway

**Fig 5:**
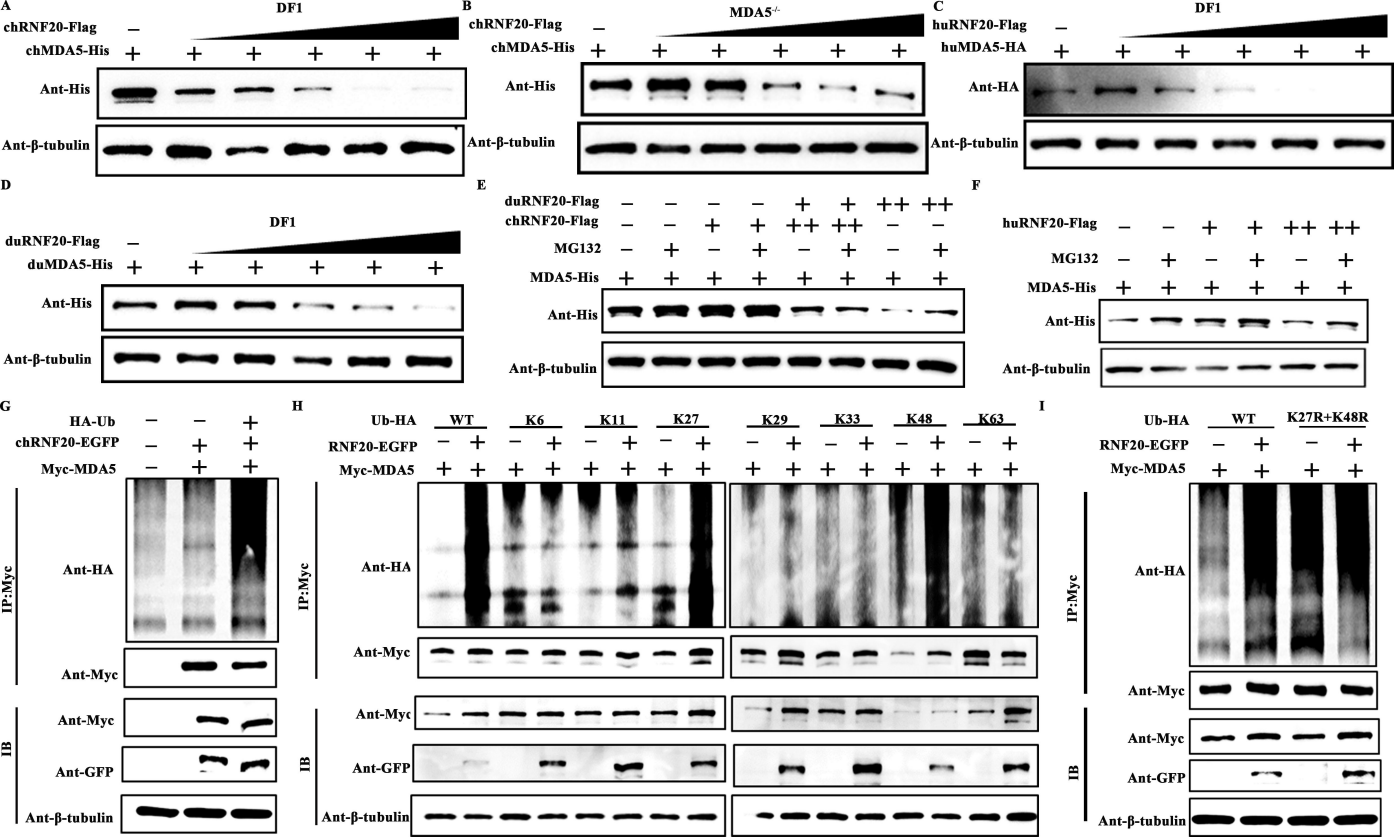
RNF20 degrades MDA5 via the ubiquitin–proteasome pathway. (A and B) Immunoblot analysis of the expression of His-tagged MDA5 and β-tubulin in DF-1 or DF-1 MDA5^−/−^ cells transiently transfected with MDA5 expression plasmid (500 ng/well) and along with increasing amounts (wedge) of chicken RNF20-expressing plasmid (0, 50, 100, 150, 200, and 250 ng/well). The cells were infected with NDV (multiplicity of infection [MOI] =1) for 24 h. (C and D) Immunoblot analysis of the expression of His-tagged MDA5 and β-tubulin in DF-1 cells transiently transfected with human or duck MDA5 expression plasmid (500 ng/well) along with increasing amounts (wedge) of human or duck RNF20-expressing plasmid (0, 50, 100, 150, 200, and 250 ng/well). The cells were infected with NDV (multiplicity of infection [MOI] =1) for 24 h. (E and F) Immunoblot analysis of the expression of His-tagged MDA5 and β-tubulin in DF-1 cells transiently transfected with chicken MDA5 expression plasmid (500 ng/well) along with chicken or human or duck RNF20-expressing plasmid (250 and 500 ng/well) then treated or untreated with MG132 (10 mM) for 4 h. (**G)** HEK293T cells were transfected with Myc-MDA5 (1,000 ng/well) and HA-ubiquitin (HA-Ub) (500 ng/well) expression plasmid, as well as a control vector or RNF20-EGFP (1,000 ng/well) in six-well plates. Co-immunoprecipitation (Co-IP) with cell lysates was performed after 12 h using Myc antibodies, followed by immunoblotting (IB). (**H)** HEK293T cells were transfected with Myc-MDA5 (1,000 ng/well), RNF20-EGFP (1,000 ng/well) and HA-ubiquitin (WT), HA-ubiquitin (K6), HA-ubiquitin (K11), HA-ubiquitin (K27), HA-ubiquitin (K29), HA-ubiquitin (K33), HA-ubiquitin (K48), or HA-ubiquitin (K63) (500 ng/well) expression plasmid in six-well plates. Co-IP with cell lysates was performed after 12 h using Myc antibodies, followed by immunoblotting (IB). (**I)** HEK293T cells were transfected with Myc-MDA5 (1,000 ng/well), RNF20-EGFP (1,000 ng/well), HA-ubiquitin (WT), or HA-ubiquitin (K27R + K48R) (500 ng/well) expression plasmid in six-well plates.

To further demonstrate that RNF20 inhibits the MDA5-mediated innate immune response, we co-transfected MDA5 and RNF20 into DF-1 wild-type or MDA5^−/−^ cell lines. The results showed that RNF20 reduced MDA5 protein expression in a concentration-dependent manner (Fig. 5A and B). Additionally, RNF20 from humans and ducks also reduces MDA5 protein expression (Fig. 5C and D), suggesting that RNF20 negatively regulates the innate immune response by modulating MDA5 protein levels, a mechanism conserved across species.

As a member of the RING family E3 ubiquitin ligase, RNF20 catalyzes the ubiquitination and subsequent degradation of substrate proteins. To confirm that RNF20 induces MDA5 ubiquitination and degradation, we used the ubiquitin–proteasome inhibitor MG132 following the co-transfection of RNF20 and MDA5. MG132 effectively inhibited the degradation of MDA5 by RNF20 (Fig. 5E and F), supporting the involvement of the ubiquitin–proteasome pathway in RNF20-mediated MDA5 degradation.

Further, we assessed the effects of RNF20 on MDA5 ubiquitination through co-immunoprecipitation assays, which showed that RNF20 overexpression enhanced MDA5 ubiquitination (Fig. 5G). To determine the type of ubiquitination catalyzed by RNF20, we co-transfected RNF20 and MDA5 with different ubiquitination plasmids. RNF20 catalyzes K27- and K48-linked polyubiquitination of MDA5 (Fig. 5H). Additional ubiquitination assays using a double-mutant ubiquitin (K27R + K48R) showed that MDA5 was ubiquitinated by RNF20 only in the presence of wild-type ubiquitin, not the K27R + K48R mutant. These findings establish that RNF20 facilitates K27/48-linked polyubiquitination and degradation of MDA5.

### RNF20 knockout *in vitro* leads to immunosuppression

**Fig 6:**
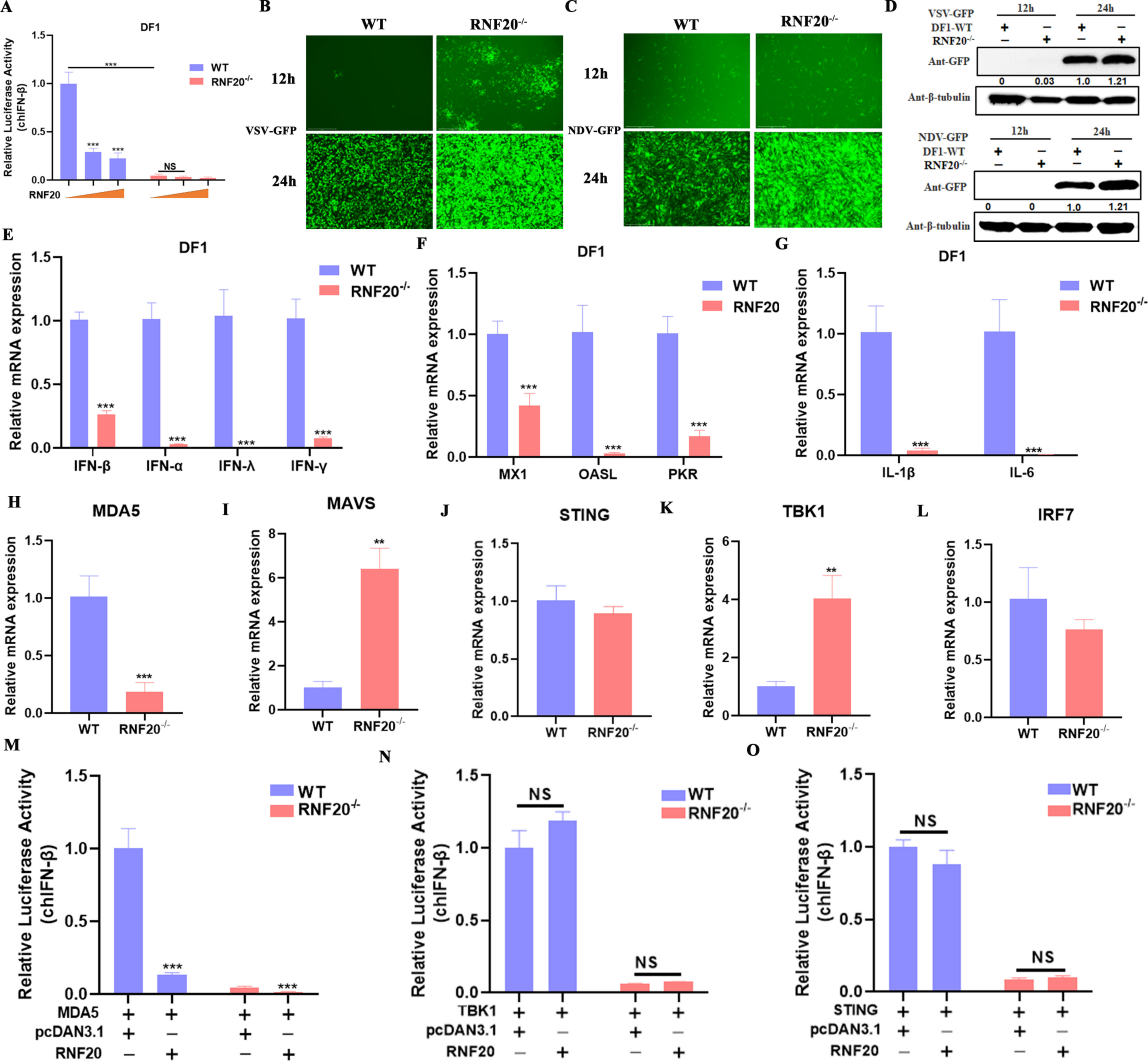
RNF20 knockout *in vitro* leads to immunosuppression. (A) DF-1 cells or DF-1 RNF20^−/−^ cells were transiently transfected with RNF20-expressing plasmid (0, 100, and 200 ng/well), pRL-TK (60 ng/well), and IFN-β Luc (120 ng/well). After 24 h, the cells were infected with VSV-GFP (multiplicity of infection [MOI] =1) for 12 h and analyzed by dual luciferase reporter. (B and C) Microscopy imaging showing VSV-GFP (multiplicity of infection [MOI] =1) or NDV-GFP (multiplicity of infection [MOI] =1) infection in DF-1 (WT) and DF-1 (RNF20^−/−^) cells at 12 and 24 h. (**D)** Immunoblot analysis of the expression of GFP-tagged proteins or β-tubulin in DF-1 cells or DF-1 RNF20^−/−^ cells infected with VSV-GFP or NDV-GFP for 12 and 24 h. (E-L) DF-1 cells or DF-1 RNF20^−/−^ cells infected with VSV-GFP for 24 h. The cells were harvested for detecting mRNA expression of type I interferons (IFN-α and IFN-β), type II interferons (IFN-γ), and type III interferons (IFN-λ), OASL, MX1, PKR, MDA5, MAVS, STING, TBK1, and IRF7. (**M-O)** DF-1 cells or DF-1 RNF20^−/−^ cells were transiently transfected with MDA5, TBK1, or STING-expressing plasmid (100 ng/well) along with RNF20-expressing plasmid or control plasmid (150 ng/well), pRL-TK (60 ng/well), and IFN-β Luc (120 ng/well). After 24 h, the cells were infected with VSV-GFP (multiplicity of infection [MOI] =1) for 12 h and analyzed by dual luciferase reporter. Data from three independent experiments are shown as mean ± SD. *P < 0.05, **P < 0.01, and ***P < 0.001 are considered statistically significant. Scale bars represent 100 µm.

To investigate the role of RNF20 in regulating the chicken innate immune response, we utilized CRISPR-Cas9 technology to generate an RNF20 knockout DF-1 cell line. Surprisingly, RNF20 knockout significantly impaired IFN-β promoter activation compared with wild-type cells (Fig. 6A). We then infected both wild-type and RNF20^−/−^ DF-1 cells with RNA viruses, such as VSV-GFP and NDV-GFP. The results showed that RNF20^−/−^ cells exhibited markedly stronger viral fluorescence than wild-type cells (Fig. 6B and C). Western blot analyses confirmed that NDV-GFP and VSV-GFP viral protein levels were higher in RNF20^−/−^ cells compared with wild-type cells (Fig. 6D). These findings suggest that RNF20 knockout leads to suppressed innate immune responses and increased viral replication.

To further demonstrate the suppression of the innate immune response in RNF20^−/−^ cells, we assessed the expression of innate immune and inflammatory factor-related genes by qPCR. RNF20 knockout significantly reduced the expression of innate immune genes, such as IFN-α, IFN-β, IFN-γ, IFN-λ, OASL, MX1, and PKR, as well as inflammatory factors, like IL-1β and IL-6 (Fig. 6E through G). Analysis of the RLR signaling pathway revealed that RNF20 knockout specifically inhibited MDA5 expression, without affecting MAVS, STING, TBK1, and IRF7 expression (Fig. 6H through L). These results indicate that RNF20 is crucial for maintaining MDA5 expression.

Furthermore, we co-transfected RNF20 with RLR signaling pathway molecules (MDA5, STING, or TBK1) into DF-1 wild type, and RNF20^−/−^ cells showed that RNF20 specifically inhibited MDA5-induced IFN-β promoter activation, while not affecting TBK1 and STING-induced activation in either cell type (Fig. 6M and O). This suggests that RNF20 dually regulates MDA5-mediated innate immune response.

### Conditional knockout of RNF20 *in vivo* inhibits MDA5-mediated innate immune response

**Fig 7:**
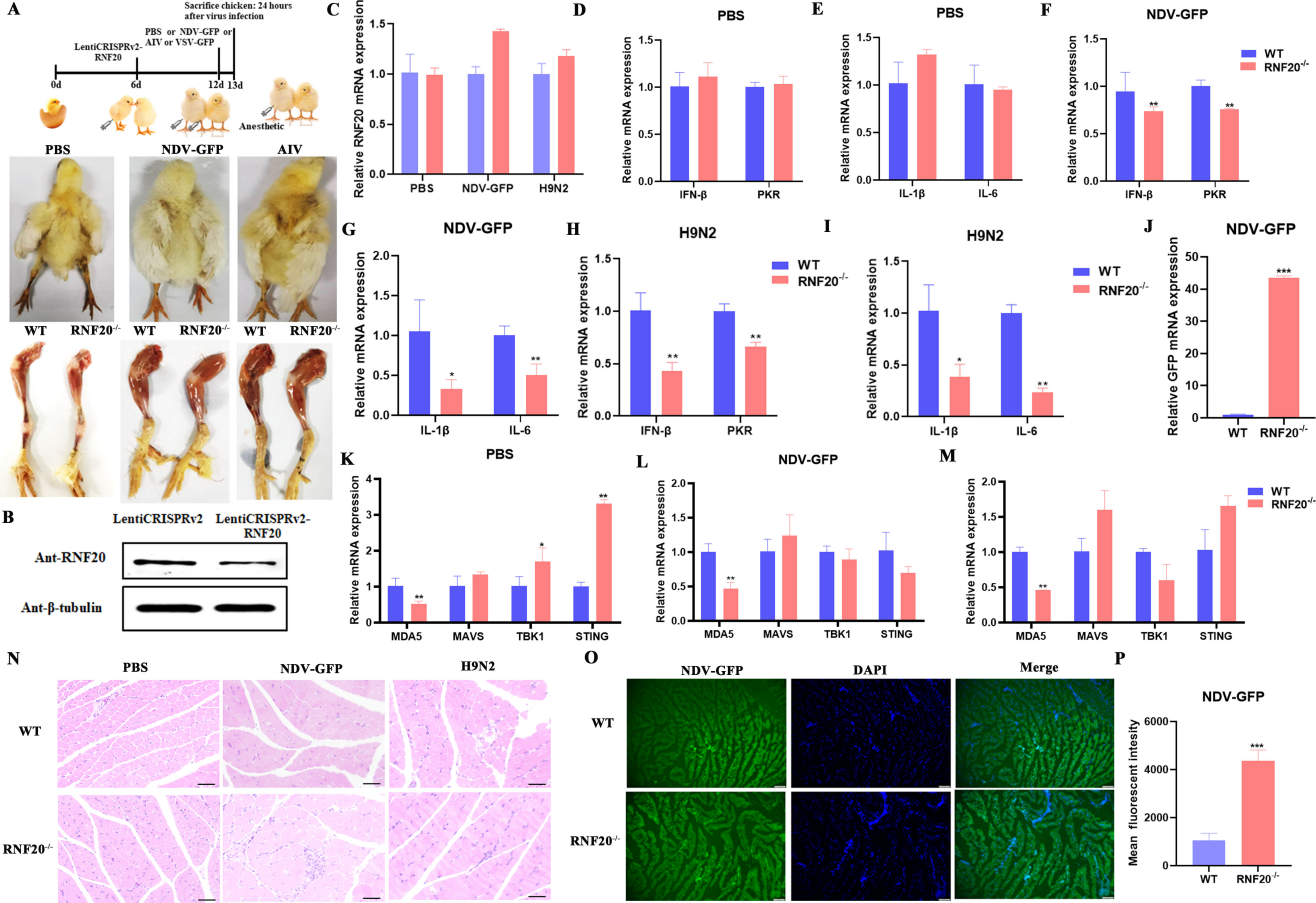
Conditional knockout of RNF20 *in vivo* inhibits MDA5-mediated innate immune response. (A) Flow chart of 1-week-old chicken experiment: the left leg was injected with lentivirus control group (Lenti-CRISPR-V2), and the right leg was injected with RNF20 knockout lentivirus (Lenti-CRISPR-RNF20) and divided into three groups according to body weight and gender after injecting for 6 days. They were then injected with PBS or NDV-GFP or AIV for 24 h, euthanized under anesthesia (upper layer), and leg muscle tissues were collected (lower layer). (**B)** Immunoblot analysis of RNF20 or β-tubulin in the chicken leg muscles after injecting lentiCRISPR-V2 or lentiCRISPR-RNF20 (5 × 10^7^ IU/chicken) for 6 days. (**C-I)** mRNA expression of RNF20, IFN-β, PKR, IL-1β, and IL-6 in the leg muscles of wild-type (WT) or RNF20^−/−^ chickens after injection with PBS, NDV-GFP, or AIV for 24 h. (**J)** mRNA copy number of NDV-GFP in the leg muscles of wild-type (WT) or RNF20^−/−^ chickens after injection with NDV-GFP for 24 h. (**K-M)** mRNA expression of MDA5, MAVS, TBK1, and STING in the leg muscles of wild-type (WT) or RNF20^−/−^ chickens after injection with PBS, NDV-GFP, or AIV for 24 h. (**N)** Hematoxylin and eosin (H&E) staining of chicken leg muscles from wild-type (WT) or RNF20^−/−^ chickens after injection with PBS, NDV-GFP (10⁵ EID50), or H9N2 (10⁴ EID50) for 24 h. (**O)** Microscopy imaging showing frozen sections of wild-type and RNF20^−/−^ chicken leg muscles after injection with NDV-GFP for 24 h. Green fluorescence represents NDV-GFP, and blue indicates the nucleus. (**P)**mage J analysis of NDV-GFP green fluorescence intensity in chicken leg muscles. Data from three independent experiments are shown as mean ± SD. *P < 0.05, **P < 0.01, and ***P < 0.001 are considered statistically significant.

To further investigate the role of RNF20 in the innate immune response, we used a lentiviral knockout system to target RNF20 in the leg muscles of 1-week-old chickens. The chickens were then injected with PBS, NDV-GFP, or H9N2 into the legs. At 24 h post-infection, the chickens were anesthetized and euthanized (Fig. 7A). The results showed that the lentiviral vector Lenti-CRISPRV2-RNF20 significantly reduced RNF20 protein levels (Fig. 7B) but did not affect RNF20 mRNA expression (Fig. 7C).

Subsequently, we performed qPCR to analyze the expression of genes related to innate immunity and inflammation in both wild-type (WT) and RNF20 knockout (RNF20^−/−^) groups. In the PBS treatment group, there were no differences in the expression of innate immunity-related genes IFN-β and PKR or inflammation-related genes IL-1β and IL-6 (Fig. 7D and E). However, following treatment with NDV-GFP and H9N2, these gene expressions were significantly reduced in the RNF20 knockout group (Fig. 7F through I), leading to extensive viral replication (Fig. 7G). This indicates that RNF20 knockdown impairs the innate immune response to RNA viral infection.

We also examined the RLR signaling pathway and found that RNF20 knockout significantly suppressed both basal and inducible MDA5 expression, without affecting MAVS, TBK1, and STING mRNA levels (Fig. 7E through M). Hematoxylin–eosin staining revealed increased immune cell infiltration and muscle damage in RNF20 knockdown chickens compared with WT controls after NDV-GFP or H9N2 infection (Fig. 7N). Additionally, the staining of frozen muscle tissue sections showed significantly higher NDV-GFP fluorescence intensity in the RNF20 knockout group (Fig. 7O and P). These results suggest that RNF20 knockdown impairs MDA5-mediated innate immune responses.

### RNF20 nucleoplasmic translocation dually regulates MDA5-mediated innate immune response during viral infection

**Fig 8:**
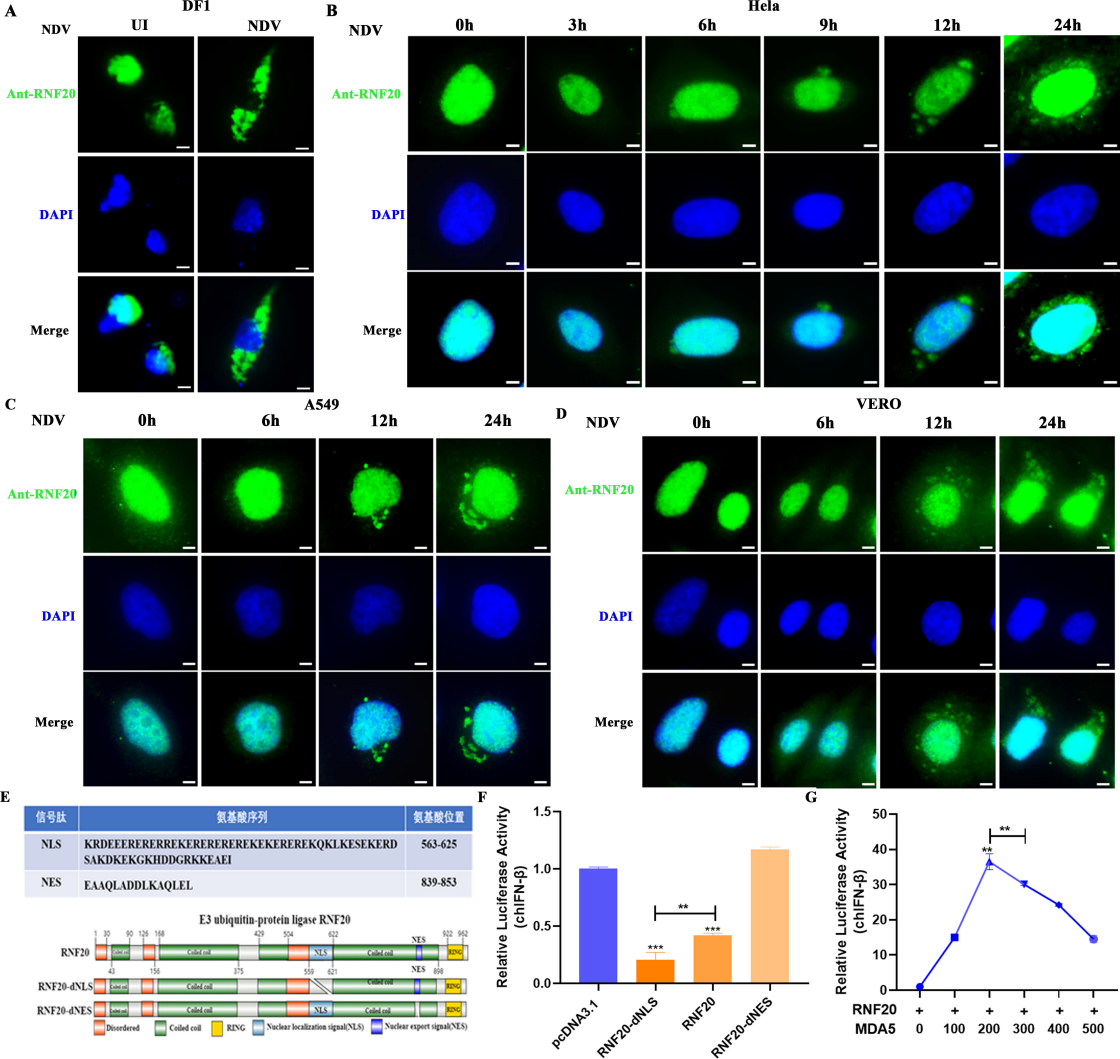
RNF20 nucleoplasmic translocation dually regulates MDA5-mediated innate immune response during viral infection. (A) Confocal microscopy images of chicken DF-1 cells transfected with RNF20-EGFP plasmid or empty vector (500 ng/well), then uninfected or infected with NDV for 12 h. (**B-D)** Confocal microscopy images of HeLa, A549, and Vero cells, uninfected or infected with NDV for 3, 6, 12, and 24 h, followed by staining with RNF20-specific primary antibody and Alexa Fluor 488-conjugated goat anti-rabbit IgG (green). (**E)** Schematic diagram of RNF20, including its nuclear localization signals (NLS) and nuclear export signals (NES), and truncated mutants. (**F)** DF-1 cells were transiently transfected with RNF20, RNF20-dNES, and RNF20-dNLS expressing plasmid, or control plasmid (500 ng/well), pRL-TK (60 ng/well), and IFN-β Luc (120 ng/well). After 24 h, the cells were infected with NDV (multiplicity of infection [MOI] =1) for 12 h and analyzed by dual luciferase reporter. (**G)** DF-1 cells were transiently transfected with RNF20-expressing plasmid (100 ng/well) along with different concentrations of MDA5-expressing plasmid (0, 100, 200, 300, 400, and 500 ng/well), pRL-TK (60 ng/well), and IFN-β Luc (120 ng/well). After 24 h, the cells were infected with NDV (multiplicity of infection [MOI] =1) for 12 h and analyzed by dual luciferase reporter. Data from three independent experiments are shown as mean ± SD. *P < 0.05, **P < 0.01, and ***P < 0.001 are considered statistically significant. Scale bars represent 10 µm.

In this study, we demonstrate that RNF20 dually regulates the MDA5-mediated innate immune response, primarily involving its nucleoplasmic function. To investigate the underlying mechanisms, we transfected chicken DF-1 cells with the RNF20-EGFP plasmid and subsequently infected them with the NDV virus. In uninfected cells, RNF20 fluorescence was predominantly localized in the nucleus; however, NDV infection significantly promoted the diffusion of RNF20 into the cytoplasm (Fig. 8A). This suggests that NDV may trigger the nucleoplasmic translocation of RNF20.

To verify this hypothesis, we infected HeLa, A549, and VERO cells with NDV and subsequently observed the subcellular localization of RNF20. In uninfected cells, RNF20 was predominantly localized in the nucleus; however, viral replication resulted in a progressive shift of RNF20 to the cytoplasm (Fig. 8B through D). This implies that viral infection induces the nucleoplasmic translocation of RNF20, which might negatively regulate the innate immune response.

Nucleoplasmic translocation of proteins relies on nuclear localization signals (NLS) and nuclear export signals (NES). To further elucidate the role of RNF20 in the nucleus and cytoplasm, we predicted the NLS and NES sequences of RNF20 using online tools. We then constructed RNF20–dNLS and RNF20–dNES expression plasmids, and transfected them into DF-1 cells (Fig. 8E). The deletion of the RNF20 NLS significantly enhanced its inhibitory effect on the IFN-β promoter, whereas the deletion of the NES abolished this effect (Fig. 8F). This suggests that the cytoplasmic localization of RNF20 negatively regulates the innate immune response.

To demonstrate that viral infection triggers RNF20 to exit the nucleus and target MDA5, thereby modulating the innate immune response, we co-transfected RNF20 with MDA5 into DF-1 cells. We observed that increasing MDA5 overexpression initially led to a significant activation of the IFN-β promoter, followed by a decrease (Fig. 8G). This strongly indicates that RNF20 dually regulates the MDA5-mediated innate immune response and prevents excessive immune activation.

## DISCUSSION

During viral infections, rapid activation of the innate immune response can effectively control the infection. However, continued activation can lead to tissue and organ damage (26). Therefore, tight regulation of the initiation and termination of innate immune signaling is critical to maintaining immune homeostasis (27). Ubiquitination has been shown to play a critical role in regulating innate immune responses by controlling the translocation, activation, and interaction of signaling molecules (9). E3 ubiquitin ligase are responsible for recognizing substrate proteins and mediating various types of ubiquitination, thereby accurately regulating the activation or degradation of substrate proteins (28). The RNF family is a large family of E3 ubiquitin ligases, but the roles of its members in chicken innate immune regulation are not well understood. In this study, we found that chicken RNF20 dually regulates MDA5-mediated innate immune response.

Previous studies have demonstrated that monoubiquitination of histone H2B at lysine 120, catalyzed by human RNF20, is essential for the activation of intracellular interferon-stimulated gene (ISG) expression in response to viral infection. This indicates that RNF20 plays a critical role in maintaining the host’s innate immune response (29). However, the role of RNF20 in the chicken innate immune response remains unclear. To validate the role of RNF20, we performed knockdown and overexpression experiments. The results indicated that overexpression of RNF20 significantly inhibited the expression of chicken innate immunity-related genes and inflammatory cytokine-related genes, while knockdown of RNF20 had the opposite effect. This indicates that RNF20 negatively regulates the chicken innate immune response.

This difference in the role of chicken RNF20 compared with its function in mammals may be attributed to species-specific variations or differing roles of RNF20 in innate and adaptive immune responses. To explore this, we cloned and validated RNF20 from multiple species and observed that RNF20 from various species significantly inhibited both IFN-β promoter activation and mRNA expression. These indicate that RNF20’s function is conserved across species, with functional differences potentially specific to certain immune cells.

The RLR signaling pathway plays a crucial role in RNA viral infection (30). Although chickens naturally lack RIG-I, they can activate a strong innate immune response through the unique MDA5-STING-IFNs signaling pathway (7, 31, 32). In this study, we demonstrate that RNF20 significantly inhibited chicken IFN-β promoter activation induced by light chain and heavy chain poly (I: C). These findings suggest that RNF20 may inhibit chicken MDA5-mediated innate immune responses.

To investigate the mechanism by which RNF20 negatively regulates innate immune responses via MDA5, we performed Co-IP analysis and identified a physical interaction between RNF20 and MDA5. Furthermore, RNF20 was found to catalyze K27- and K48-linked ubiquitination, leading to the degradation of MDA5, thereby negatively regulating innate immune responses. Although numerous E3 ubiquitin ligases that catalyze the activation or degradation of MDA5 have been identified in mammals, the regulatory mechanisms of chicken MDA5 remain poorly understood (33). To date, only chicken TRIM25 has been identified as catalyzing the ubiquitination and activation of MDA5 (34). Here, we revealed that RNF20 mediates the classical K48 and non-classical K27-linked ubiquitination and degradation of chicken MDA5, thereby preventing excessive immune responses. Notably, human RNF20 can suppress IFN-γ-induced IRF1 transcription by modulating the phosphorylation of the C-terminal domain (CTD) of RNA polymerase II (35), suggesting that species-specific differences in RNF20 regulate innate immunity.

To further investigate the role of chicken RNF20 in innate immunity, we established RNF20 knockout DF-1 cell lines and conditional knockout chickens. Surprisingly, both RNF20 knockout cells and chickens exhibited immunodeficiency. Subsequent analysis revealed that RNF20 knockout significantly reduced MDA5 expression, leading to impairments in MDA5-mediated innate immune responses. This suggests that RNF20 is crucial for maintaining the innate immune response. Based on mammalian studies, RNF20 mediates chromatin modification in the nucleus, thereby regulating cell division, proliferation, and differentiation (19, 23, 36). Where histone ubiquitination is essential for chromatin relaxation and gene expression, the absence of RNF20 leads to defects in chromatin H2B modification. This suggests that chicken RNF20 may also regulate MDA5 expression through the monoubiquitination of histone H2B.

The subcellular localization of the protein is closely linked to its function (37). In this study, we found that RNF20 negatively regulates MDA5-mediated innate immune response. However, RNF20 is also critical for maintaining MDA5 expression. This involves nuclear and cytoplasmic functions. Thus, we analyzed the subcellular localization of chicken RNF20. Interestingly, RNF20 is primarily localized in the nucleus of uninfected cells, while Newcastle disease virus (NDV) infection significantly increases its cytoplasm distribution. Similarly, endogenous RNF20 in mammalian cells is predominantly nuclear, and viral replication markedly enhances its cytoplasmic distribution. Functional studies on RNF20’s nuclear and cytoplasmic roles revealed that RNF20 negatively regulates MDA5-mediated innate immune responses when localized in the cytoplasm, but loses this inhibitory function when localized in the nucleus. This suggests that RNF20 dually regulates MDA5-mediated innate immune responses depending on its localization and that viral infection can trigger the nucleocytoplasmic translocation of RNF20.

**Fig 9:**
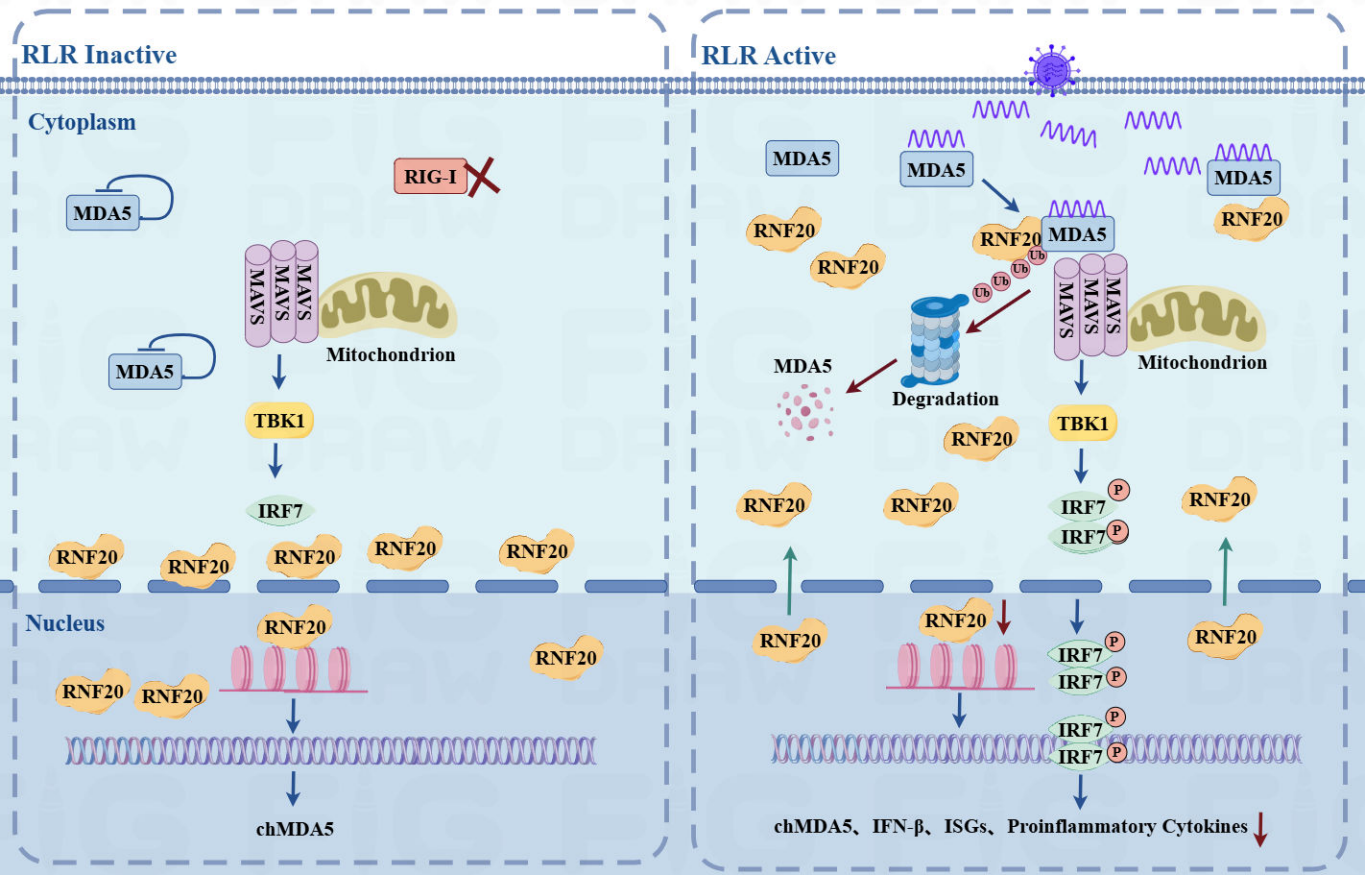
A model of RNF20 demonstrates dual regulation of the chicken MDA5-IFN-β signaling pathway by targeting chMDA5 (created using Figdraw). When the RLR signaling pathway is inactive, RNF20 maintains chMDA5 transcription in the nucleus. Upon viral infection, the RLR signaling pathway is activated, causing RNF20 to translocate from the nucleus to the cytoplasm. In the cytoplasm, RNF20 interacts with chMDA5, catalyzing its K27 and K48-linked polyubiquitination, leading to chMDA5 degradation.

In summary, our study found that viral infection triggers RNF20 to dually regulate MDA5-mediated innate immune responses, thereby maintaining innate immune homeostasis. Specifically, RNF20 regulates both basal and inducible expression of MDA5 in the nucleus to maintain immune defense, while in the cytoplasm, it catalyzes K27/48-linked ubiquitination and degradation of MDA5 to prevent excessive immune responses. Viral infection triggers a switch in the regulatory function of RNF20 through nucleoplasmic translocation (Fig. 9). Therefore, RNF20 is a critical regulator of innate immune responses.

## MATERIALS AND METHODS

### Cell culture and virus

Human HEK293T cells, A549 cells, HeLa cells, chicken DF-1 cells, and bat TB 1 Lu cells, and *Cercopithecus aethiops* VERO cells were obtained from the American Type Culture Collection (ATCC, Manassas, VA, USA). They were cultured in DMEM (Gibco, Carlsbad, CA) containing 10% FBS supplemented with 1% penicillin/streptomycin. The cells were cultured at 37°C under 5% CO2. The Newcastle disease virus was a low virulent strain of LaSota named NDV. The avian influenza virus (AIV) strain A/Chicken/Shanghai/010/2008 (H9N2) (SH010), isolated from a chicken in Shanghai, China, in 2008, was identified as H9N2 avian influenza A virus. The GFP-tagged vesicular stomatitis virus (VSV-GFP) was stored in our laboratory. All viruses were purified, propagated, and stored as previously described (38).

### Animals and treatment

Zero‐day‐old specific pathogen-free (SPF) chicken embryos were purchased from the Shanghai Academy of Agricultural Sciences. The embryos were incubated in a constant temperature and humidity incubator, with egg rotation every 12 h. The incubation conditions were as follows: days 1–7 at 38.2°C with 65% humidity; days 8–17 at 37.8°C with 70% humidity; and days 18 to hatching at 37.6°C with 80% humidity, with warm water sprayed five times daily. After hatching, the chicks were transferred to a constant temperature incubator at 37°C and given free access to water. Feeding was initiated 3 days post-hatching. All chickens were housed in negative-pressure-filtered air isolators and fed as recommended guidelines. After 1 week of adaptation, the chicks were injected with lentivirus in their leg muscles. Specifically, the left leg of each chicken was injected with the lentiCRISPR-V2 control vector, while the right leg was injected with lentiCRISPR-RNF20. One-week post-injection, the chicks were randomly divided into three groups based on weight and sex: the control group (PBS), the Newcastle disease virus (NDV) infected group, and the avian influenza virus (H9N2) infected group. The viruses were diluted in phosphate-buffered saline (PBS) and injected into the leg muscles. The AIV group was inoculated with 10⁴ EID50 of H9N2 viruses, the NDV group with 10⁵ EID50 of NDV, and the control group with 100 µL PBS. Three days post-inoculation, the chickens were euthanized using ether and subsequently killed. Leg muscle tissues were collected, immediately frozen in liquid nitrogen, and stored at −80°C for later analysis.

### Construction of plasmid

The chicken RNF family protein sequence was obtained from the National Center for Biotechnology Information (NCBI). Primers were designed based on the sequence to amplify the corresponding cDNA from DF-1 cells using RT-PCR. The PCR product was ligated into a pTOPO-Blunt vector (Vazyme Biotech co., Ltd.) for sequencing, and the positive colonies were sent to the Beijing Genomics Institute (Beijing, China) for sequencing. Then, the full-length sequence of the amplified cDNA was inserted into the HindIII and EcoRI restriction sites of the pcDNA3.1 expression vector using the Hieff Clone Plus One Step Cloning Kit (Yeasen, Shanghai, China). The recombinant plasmid was then transformed into DH5α Chemically Competent Cells (Tsingke Biology Technology, Beijing, China) for plasmid propagation.

### RNA interference

To knockdown chicken RNF20, short hairpin RNAs (shRNAs) targeting RNF20 were constructed using the pGPU6/Neo vector. The plasmids were then transferred into cells, and the knockdown efficiency was analyzed by qPCR and immunoblot analysis. The specific shRNA sequences used in this study are as follows: shRNF20#1: 5′-GAAGCACCGAATAATGTCTCA-3′ shRNF20#2: 5′-GACATGCAGGAGCAGAACATA-3′

### CRISPR-Cas9–mediated genome editing

To generate DF-1 RNF20 knockout cell lines, CRISPR-Cas9 gene-editing technology was employed. Guide sequences targeting RNF20 were cloned into the PX459 vector (Addgene). DF-1 cells were transfected with the PX459 vector, and stable clones were selected using puromycin (2 µg/mL) for 5 days. The resulting clones were isolated, and the expression of the target gene was confirmed by Western blot analysis.

For generating RNF20 knockout chickens, the lenti-CRISPRv2 vector (Addgene 52961) and lentiviral packaging plasmids (pMD2.G [Addgene 12259] and psPAX2 [Addgene 12260]) were used. Guide sequences targeting RNF20 were annealed and cloned into the lenti-CRISPRv2 vector. The plasmid construction was confirmed by sequencing.

Lentiviral particles were produced by co-transfecting HEK293T cells with the CRISPRv2-chRNF20 plasmid and the packaging plasmids (pMD2.G and psPAX2) at a ratio of 1:1.5:1.5. The supernatant was collected 48 h post-transfection and filtered. The packaged lentivirus was then used to infect chicken cells with polybrene (1:1,000 dilution). The guide sequence used to target RNF20 was as follows: 5′-ACAGTGGAAACAATTAAGCT-3′.

### Cell transfection

Cells were seeded in 24-, 12-, or 6-well plates (NEST Biotechnology, Wuxi, China) at densities of 5 × 10^5 cells/mL for 24- and 12-well plates, and 1 × 10^6 cells/mL for 6-well plates. Once the cell density reached 90%–100% confluence, transfection was performed using the Nulen PlusTrans Transfection Reagent (Nulen, Shanghai, China) following the manufacturer’s protocol.

### Ubiquitination assay

To analyze the ubiquitination of chMDA5 in HEK293T cells, cells were transfected with plasmids expressing Myc-MDA5, HA–ubiquitin (WT), HA-ubiquitin (K6), HA-ubiquitin (K11), HA-ubiquitin( K27), HA-ubiquitin (K29), HA-ubiquitin (K33), HA-ubiquitin (K48), HA-ubiquitin (K63), or HA-ubiquitin (K27R + K48R) along with RNF20-EGFP. After transfection, whole-cell extracts were immunoprecipitated using a Myc-specific antibody, and the samples were analyzed by immunoblotting with anti-HA antibody to detect ubiquitination.

### Immunofluorescence and confocal microscopy

DF-1 cells, A549 cells, Hela cells, and VERO cells were seeded in confocal dishes for imaging. The cells were fixed with 4% paraformaldehyde for 30 min, permeabilized using 0.1% Triton X-100 for 10 min, and blocked with 5% BSA in PBS for 1.5 h. After blocking, the cells were stained with specific primary antibodies. For DF-1 cells, anti-RNF20 antibody was used, and the primary antibodies were detected using Alexa Fluor 488-conjugated goat anti-rabbit IgG (green). The nuclei were stained with DAPI. Colocalization was analyzed using an Olympus IX83 confocal microscope (Olympus Corp, Tokyo, Japan), and the images were processed and analyzed using ImageJ software.

### Immunoblotting and co-immunoprecipitation (co-IP)

For immunoblot analysis, cells were lysed with RIPA Lysis Buffer (50 mM Tris [pH 7.4], 150 mM NaCl, 1% Triton X-100, 1% sodium deoxycholate, 0.1% SDS, and sodium orthovanadate, sodium fluoride, EDTA, and leupeptin) supplemented with a protease inhibitor “cocktail.” For immunoprecipitation (IP), whole-cell extracts were collected 36 h after transfection and were lysed in IP buffer (50 mM Tris [pH 7.4], 150 mM NaCl,1% NP-40, 0.5% sodium deoxycholate, 0.1% SDS, and sodium orthovanadate, sodium fluoride, EDTA, and leupeptin) supplemented with a protease inhibitor cocktail (Merck). After centrifugation for 10 min at 14,000×*g*, supernatants were collected and incubated with protein G plus Magnetic Beads Immunoprecipitation reagent together with 1 µg corresponding antibodies. After 6 h of incubation, beads were washed five times with TBST buffer. Immunoprecipitates were eluted by boiling with 1% (wt/vol) SDS sample buffer. For immunoblot analysis, immunoprecipitates or whole-cell lysates were loaded and subjected to SDS-PAGE, transferred onto nitrocellulose membranes, and then blotted with indicated Antibodies.

### Luciferase assay

HEK293T cells or DF-1 cells or TB 1 Lu were cotransfected with IFN-β luciferase reporter, pRL-TK, and other expression vectors where indicated. Luciferase activity was measured with the Dual-Luciferase Reporter Assay system according to the manufacturer’s instructions (Promega). Data were normalized for transfection efficiency by calculating the ratio between firefly luciferase activity and Renilla luciferase activity.

### RT-qPCR analysis

Total RNA was extracted from cells using AG RNAex Pro Reagent (Accurate Biology, Hunan, China), and the single-strand cDNA was reverse-transcribed with HiScript III RT SuperMix for qPCR (+gDNA wiper) (Vazyme Biotech co., Ltd.). Gene expression was examined with the ABI 7500 system by a fast two-step amplification program with AceQ Universal SYBR qPCR Master Mix (Vazyme Biotech Co.,ltd). The value obtained for each gene was normalized to that of the gene encoding β-actin. The sequences of primers used in this study are included in Table S1.

### RNA sequencing

Total RNA was extracted using the Total RNA Extractor kit (B511311, Sangon, China). A total amount of 1 µg RNA per sample was used as input material for the RNA sample preparations. Sequencing libraries were generated using VAHTSTM mRNA-seq V2 Library Prep Kit for Illumina following manufacturer’s recommendations, and index codes were added to attribute sequences to each sample. The libraries were then quantified and pooled. Paired-end sequencing of the library was performed on the NovaSeq sequencers (Illumina, San Diego, CA).

### Expression analysis

Gene expression values of the transcripts were computed by StringTie. The transcripts per million (TPM), eliminates the influence of gene lengths and sequencing discrepancies to enable direct comparison of gene expression between samples. DESeq2 was used to determine differentially expressed genes (DEGs) between two samples. Genes were considered as significant differentially expressed if q-value ≤0.001 and |FoldChange| ≥ 2. When the normalized expression of a gene was zero between two samples, its expression value was adjusted to 0.01 (as 0 cannot be plotted on a log plot). If the normalized expression of a certain gene in two libraries was all lower than 1, further differential expression analysis was conducted without this gene.

### Hematoxylin and eosin (H&E) staining

Chicken leg muscles were fixed with 4% paraformaldehyde after paraffin embedded, cut into 5 μm thick sections, deparaffinized and dehydrated, and then stained with H&E.

### Statistical analysis

All data are presented as mean ± SD of three or more experiments. GraphPad Prism 8.0 was utilized to graph the results. Data were determined by Student’s *t*-test (where two groups of data were compared). *P* values < 0.05 were considered statistically significant.

## Data Availability

The data generated or analyzed during this study are available from the corresponding author upon reasonable request.

## SUPPLEMENTAL MATERIAL

Table S1 jvi.02008-24-s0001.docx Primers used to detect relative gene mRNA expression levels with quantitative RT-PCR.

ASM does not own the copyrights to Supplemental Material that may be linked to, or accessed through, an article. The authors have granted ASM a non-exclusive, world-wide license to publish the Supplemental Material files. Please contact the corresponding author directly for reuse.
