# Supplementary material for: RNF20 dual regulation of MDA5 signaling to maintain immune homeostasis in chickens
Source: J Virol. 2025 Feb 25;99(3):e02008-24. doi: 10.1128/jvi.02008-24 (PMC11915864; doi:10.1128/jvi.02008-24)
Supplement: Table S1 — Primers used to detect relative gene mRNA expression levels with quantitative RT-PCR. [file jvi.02008-24-s0001.docx]

**Supplemental table 1:**

Primers used to detect relative genes mRNA expression levels with quantitative RT-PCR.

| **Genes** | **Primer** | **Nucleotide sequence of primers (5’-3’)** |
| --- | --- | --- |
| qchIFNβ | Forward | TCCTACTGCTCTTGCTTCTGC |
|  | Reverse | TGGAAATGGAAAAGTCACGTC |
| qhuIFNβ | Forward | GTCACTGTGCCTGGACCATAG |
|  | Reverse | GTTTCGGAGGTAACCTGTAAGTC |
| qchIFNα | Forward | ATGCCACCTTCTCTCACGAC |
|  | Reverse | AGGCGCTGTAATCGTTGTCT |
| qchIFNγ | Forward | TTCGATGTACTTGGAAATGC |
|  | Reverse | TTGCATCTCCTCTGAGACTG |
| qbatIFNβ | Forward | GCACCGGCTGGAATGAGACCA |
|  | Reverse | GTCCAGGCATTGGCTGT |
| qchPKR | Forward | TGCTTGACTGGAAAGGCTACT |
|  | Reverse | TCAGTCAAGAATAAACCATGTGTG |
| qchMX1 | Forward | GTTTCGGACATGGGGAGTAA |
|  | Reverse | GCATACGATTTCTTCAACTTTGG |
| qchIL-1β | Forward | GCTCTACATGTCGTGTGTGATGAG |
|  | Reverse | TGTCGATGTCCCGCATGA |
| qchIL-6 | Forward | AGGACGAGATGTGCAAGAAG |
|  | Reverse | TGCTGTAGCACAGAGACTCG |
| qchMDA5 | Forward | TGAAAGCCTTGCAGATGACTTA |
|  | Reverse | GCTGTTTCAAATCCTCCGTTAC |
| qchSTING | Forward | GGTCCTACTACATCGGCTACCTGA |
|  | Reverse | GGCCTGAGCTTGTTGTCCTTATCT |
| qchTBK1 | Forward | GTTTGCTATTGAGGAAGAGACAAC |
|  | Reverse | CCATTTTCCCGGAGATGATTCATC |
| qchIRF7 | Forward | GCCTGAAGAAGTGCAAGGTC |
|  | Reverse | CTCTGTGCAAAACACCCTGA |
| qchRNF20 | Forward | AGCTGCAAGAGCGTGTGGAAT |
|  | Reverse | CCACTATTCAGCTTGTGTGAC |
| qNDV-NP | Forward | TGCAGCAATGGTACTCCGTT |
|  | Reverse | CCTTTGCTACCGTGACCCAT |
| qhuβ-actin | Forward | TTTTGGCTATACCCTACTGGCA |
|  | Reverse | CTGCACAGTCGTCAGCATATC |
| qchβ-actin | Forward | CAGACATCAGGGTGTGATGG |
|  | Reverse | TCAGGGGCTACTCTCAGCTC |
| qbatβ-actin | Forward | CCATCCTGCGTCTGGACCTGG |
|  | Reverse | GTGGCCATCTCCTGCTCGAAG |
